# 

**Published:** 1914-04-11

**Authors:** 


### CONTENTS.

| | PAGE |
|---|---|
| **Westminster Hospital and Clapham Common** | 29 |
| **The Status of Institutional Dispensers** ... ... | 30 |
| **Hospital and Institutional News** ... ... ... | 31 |

Our Prize Scheme for Equipment "Tips"—A Mental Hospital Staff on Strike—The Hospital Position in Ulster—A General Post Among London Hospital Secretaries—The Staff Difficulty in Infectious Hospitals—Combination in the Fever Hospital Service — A Hospital Matron's Finance—The First Legacy for Fourteen Years—Waking up the Annual Meeting—The Annual Meeting as an Event in Itself—The Late Mr. Bruce Clarke—Retrograde Proposal by the Dental Profession—Visiting Dentists for Poor-Law Infirmaries—The Old Manchester Royal Infirmary Site—Mr. Walter Carnt's Recovery—The Insurance Act and Hospital Staffs—The London Mental Deficiency Committee—The Travelling Expenses of Sanatoria Patients—The Attendance of Medical Officers at Guardians' Meetings—Hours and Salaries of Public Ophthalmic Medical Officers—The Infirmary Movement in Wales—Government by General Committee—A Pharmacist's Path to Fame—The New Sanatorium at Derby—The Doctor's Wife—Easter and the Drug Market.

| | PAGE |
|---|---|
| **Common Errors in Diagnosis** ... ... ... | 37 |
| The Skin. | |
| **Research and Remedies** ... ... ... ... | 38 |
| **The Medical Aspects of Aviation** ... ... ... | 39 |
| The Results of Flight upon Normal Health. | |
| **Some Dangers of Drug Habits** ... ... ... | 40 |
| The Effects of Drugging in Life and in Literature. | |

[*See over.*]

### CONTENTS—(*continued*).

| | PAGE |
|---|---|
| **Domestic Control in Little Hospitals** ... ... | 41 |
| A Workable System of Cottage Hospital Book-Keeping. | |
| **Buildings Contemplated and in Progress** ... | 42 |
| **Hospitals from the Patients' Point of View** ... | 43 |
| Varied Experiences in a Surgical Ward. | |
| **Vegetable Soups in the Treatment of Children's Anæmias** ... ... ... ... ... | 44 |
| **Relaxations and Hobbies** ... ... ... ... | 45 |
| Wild Life in a Hospital Barrack-Square. | |
| **Round the World** ... ... ... ... ... | 46 |
| Fellow-Workers and their Doings. | |
| **National Insurance Act Developments** ... ... | 47 |
| The Interim Report of the Fabian Society (*concluded*). | |
| **The Origin and Evolution of the 18th Century Hospital Movement** (*concluded.*) ... ... ... | 51 |
| **The Editor's Letter-Box** ... ... ... ... ... | 49 |
| Salaries of Poor-Law Medical Officers. | |
| Women Doctors and the Ban on Marriage. | |
| A Simple Means of Making Carbon Dioxide Snow. | |
| **The Doctors' Wives Association** ... ... ... | 52 |
| **The Relation of Clothes to Appetite** ... ... | 53 |
| Overfeeding and Hygienic Management. | |
| **The National Medical Union (Official)** ... ... | 51 |
| Resolutions on Policy. | |
| The Profession of Medicine. | |
| **The Institutional Worker** ... ... (Suppl.) | 1 |
| Hospital Washing. | |
| Questions for April. | |
| The Filing of Out-patient Case-papers. | |
| The Sick and in Need. | |
| Employment and Training. | |

